# The Emerging Role of GC-MSCs in the Gastric Cancer Microenvironment: From Tumor to Tumor Immunity

**DOI:** 10.1155/2019/8071842

**Published:** 2019-12-02

**Authors:** Zhaoji Pan, Yiqing Tian, Guoping Niu, Chengsong Cao

**Affiliations:** ^1^Xuzhou Central Hospital, The Affiliated Xuzhou Hospital of Medical College of Southeast University, Xuzhou, Jiangsu, China; ^2^Xinyi People's Hospital, Xinyi, Xuzhou, Jiangsu, China

## Abstract

Mesenchymal stem cells (MSCs) have been declared to not only participate in wound repair but also affect tumor progression. Tumor-associated MSCs, directly existing in the tumor microenvironment, play a critical role in tumor initiation, progression, and development. And different tumor-derived MSCs have their own unique characteristics. In this review, we mainly describe and discuss recent advances in our understanding of the emerging role of gastric cancer-derived MSC-like cells (GC-MSCs) in regulating gastric cancer progression and development, as well as the bidirectional influence between GC-MSCs and immune cells of the tumor microenvironment. Moreover, we also discuss the potential biomarker and therapeutic role of GC-MSCs. It is anticipated that new and deep insights into the functionality of GC-MSCs and the underlying mechanisms will promote the novel and promising therapeutic strategies against gastric cancer.

## 1. Introduction

Mesenchymal stem cells (MSCs), known as mesenchymal stromal cells as well, were first discovered in pig bone marrow stroma in 1960s and identified as fibroblast colonies [1]. Then, they were found to exist in most tissues and play significantly important roles in several types of disease, including inflammatory diseases, tissue regeneration healing, and organ injury diseases [2–11]. MSCs have the plasticity characteristic, which means they could not only enhance tissue healing and promote immune responses but also have the inhibitory function, according to the pathophysiological status of the tissue where they reside [12, 13]. Recently, MSCs have been found to affect tumor progression and function as key regulators of tumor fate [9, 14–17]. And MSCs derived from different tumor types could influence tumor progression through different mechanisms. Tumor-associated MSCs (TA-MSCs) from ovarian cancer or multiple myeloma were reported to promote tumor growth by secreting some growth factors or exosomes [18, 19]. In a human colorectal cancer xenograft model, TA-MSCs could promote tumor angiogenesis in an IL-6- and endothelin-1-dependent way, whereas CAFs and normal fibroblasts could not [20]. Moreover, TA-MSCs in breast cancer enhanced the motility, invasive ability, and metastasis of tumor cells by CCL5/CCR5 signaling axis [21]. So, TA-MSCs are distinctively unique in different tumor types.

The tumor microenvironment (TME) is the complex microenvironment composed of different cellular types including tumor cells, endothelial cells, stromal cells, and immune cells [22, 23]. Tumors are considered to be wounds which do not heal [24], and MSCs were reported to have the immunosuppressive functionality [25]. Recently, many studies have demonstrated that MSCs could affect the phenotype and functionality of T cells including mediating the CD4^+^ T cell migration and differentiation [26], modulating T helper 17/regulatory T balance [27], and controlling memory T cell responses [28]. MSCs are also involved in the immunomodulatory function of B cells, dendritic cells, macrophages, and myeloid-derived suppressor cells (MDSCs) [29–32]. So, it is easily understandable that MSCs interact with immune cells and other cells in the TME. Moreover, MSCs have been reported to influence tumor progression through regulating immune cells in different tumor types [33–36]. However, studies about the tumor immunity role of TA-MSCs are still in infancy. Gastric cancer, the leading cause of cancer-related death worldwide, is highly concerned [37–41]. Emerging evidence demonstrated that the tumor microenvironment cells including macrophages, T cells, and fibroblasts all play critical roles in GC development and prognosis [42–45]. In this review, we mainly detail and discuss current advances in the understanding of the important role of gastric cancer-derived MSC-like cells (GC-MSCs) in gastric cancer (GC) progression. We would elaborate from how GC-MSCs interact with tumor cells to interacting with immune cells and how their interactions impact tumor progression, which is greatly meaningful for gastric cancer immunotherapy.

## 2. GC-MSCs

### 2.1. The Origin of GC-MSCs

In 2004, Studeny et al. found that bone-marrow-derived MSCs (BM-MSCs) could recruit to tumors after the intravenous injection of MSCs [46], which laid the foundation for later MSC-associated studies. In 2012, Ren et al. furtherly verified that the intrabone injection-derived green fluorescent protein (GFP)^+^ BM-MSCs could actively recruit to tumors [47]. Surprisingly, they also proved that tumor-resident MSCs are derived from BM-MSCs, revealing that BMMSCs maybe the precursors of TA-MSCs. In 2014, Ren et al. continue to demonstrate that lymphoma-resident MSCs endowed BM-MSCs with tumor-promoting properties [48], indicating that TA-MSCs could transfer BM-MSCs into TA-MSCs to expand their numbers. Supplementally, miR-155-5p inhibition was proved to promote the transition of BM-MSC into GC-MSC by targeting NF-*κ*B p65, in 2016 [49]. To sum up, BM-MSCs are the main origin of GC-MSCs, which is greatly critical for GC progression.

### 2.2. Isolation and Characteristics

In 2008, GC-MSCs, the main component of the TME of GC, were firstly discovered and isolated by Cao et al. [50]. Probably 15 days after plating, GC-MSCs commenced to form colonies, with long and spindle-shaped single cells, resembling the morphology of fibroblasts. The GC-MSCs also had a normal number of chromosomes that could maintain the shape and structure of MSCs after cell cycle progression. Moreover, contrary to GC cells, the GC-MSCs failed to form tumors when grafted into nude mice, indicating that GC-MSCs were normal cells but not a subset of tumor cells. GC-MSCs express stem cell-related genes including Oct-4, CD44, CD73, Nanog, Bmi1, and nucleostemin but were negative for hematopoietic markers such as CD34 and were negative for the specific endothelial antigen, CD31. And it has the ability to self-renew and differentiate into osteocytes or adipocytes, which was similar with BM-MSCs.

So far, the uniform standard for the biological characteristics and function of TA-MSCs is rare. In different tumor types, TA-MSCs were proved different new characteristics that were significantly different from BM-MSCs. In human ovarian cancers, TA-MSCs secrete obviously more bone morphogenetic proteins, which are indispensable for maintaining stem cell differentiation, than BM-MSCs [18]. In multiple myeloma, compared with BM-MSCs, TA-MSCs express lower levels of miR-15a, the tumor-suppressive microRNA (miRNA) [19]. In gastric cancer, Cao et al. demonstrated that GC-MSCs have higher proliferative potential than BM-MSCs, endowing GC-MSCs with quicker self-renew ability, which is consistent with the high expression of PCNA in GC-MSCs (most of BM-MSCs were negative for PCNA expression) [50].

In 2010, Xu et al. found and isolated the human gastric cancer adjacent noncancerous tissue-derived MSCs (hGCN-MSCs), which are paired with the GC-MSCs [51]. hGCN-MSCs and GC-MSCs are alike in the typical fibroblast-like appearance, paralleling to that of hBM-MSCs. In addition, both hGCN-MSCs and GC-MSCs were positive for CD29, CD44, CD90, and CD105, but negative for CD14, CD31, CD34, and CD45, which were the characteristic surface of MSCs. However, differences exist between them. The cumulative population of GC-MSCs is doubling the hGCN-MSCs and BM-MSCs. Compared with hGCN-MSCs, GC-MSCs showed a lower migration ability and CD44 expression, which is one of the important adhesion molecules that are responsible for cell migration or invasion [52].

## 3. GC-MSCs and Tumor

The following part will detail the interaction between GC-MSCs and gastric cancer environment, which plays a significantly important role in tumor development.

### 3.1. GC-MSC Secreted Molecules and Tumor Progression

Huang et al. found that PDGF-DD from the GC-MSC-conditioned medium (CM) could promote the proliferation and migration of tumor cells *in vitro* and *in vivo* by phosphorylating PDGFR-*β* in SGC-7901 cells [53]. And targeting the PDGF-DD/PDGFR-*β* interaction between GC-MSCs and tumor cells may provide a novel strategy for gastric cancer therapy. However, whether a molecule or a signaling pathway in GC-MSCs or other microenvironmental cells regulate the secretion of PDGF-DD were still unknown, which need to be further investigated. Moreover, another proinflammatory cytokine which was strikingly high secreted by GC-MSCs, interleukin-8 (IL-8), was reported to enhance the proliferation, migration, and proangiogenesis ability of GC cells partly by regulating the activation of Akt or Erk1/2 pathway, which also participated in immune regulation we would elucidate in the following tumor immunity part [54]. Either knocking down PDGF-DD in GC-MSCs or adding anti-IL-8 antibody reversed the tumor promoting role of hGC-MSCs *in vitro* and *in vivo*. However, whether the signaling pathways PDGF-DD and IL-8 participate in crossed connection need to be investigated. Sun et al. also demonstrated that GC-MSC-derived IL-15 could promote GC cell migration and epithelial-mesenchymal transition (EMT) by regulating STAT3 in GC cells [55]. Above all, GC-MSCs could secrete some molecules to promote tumor progression.

### 3.2. GC-MSC Expressed Molecules and Tumor Progression

miRNAs, the short noncoding RNAs, regulating posttranscriptional gene expression, act as oncogenes or tumor suppressors that are involved in tumor progression [56–58]. The role of miRNAs in TA-MSCs catches more and more attention, which is crucial for tumor progression [59–61]. Wang et al. firstly identified that miR-214, miR-221, and miR-222 are upregulated in GC-MSCs and gastric cancer tissues compared to GC-MSCs and adjacent noncancerous tissues [62]. Moreover, they also found that miR-221 inhibiting in GC-MSCs could obviously suppress the proliferation and migration of GC cells. Mechanistically, GC-MSC-CM-derived exosome carrying miR-221 could be delivered to GC cells to enhance the miR-221 expression in GC cells, which regulate the proliferative and migratory ability of GC cells. In addition, miR-374 in GC-MSCs, which were isolated from N-methyl-N′-nitro-N-nitrosoguanidine- (MNNG-) induced gastric cancer from gastritis in a rat experimental model, regulated their proliferative and migratory capabilities, which is critical for gastric carcinogenesis [63]. Zhu et al. not only found that miR-155-5p inhibition could regulate the transition of BM-MSC into GC-MSC-like cells acquiring a GC-MSC-like phenotype and function but also proved that miR-155-5p overexpression in GC-MSCs inhibited growth, migration, and invasion of GC *in vitro* and *in vivo* [49]. Those results show that miRNA in GC-MSCs is greatly important for GC progression and development.

In addition to miRNA, other molecules expressed in GC-MSCs also can regulate GC progression. We have proved that YAP expression in GC-MSCs can affect GC growth and progression *in vitro* and *in vivo.* However, the detailed mechanisms are not described [64]. Whether YAP regulate the molecule secretion in the GC-MSC-CM or regulate other microenvironmental cells to influence the GC progression and what is the downstream signaling pathway in GC cells are still needed to be further investigated.

Above all, molecules of GC-MSCs secreting and expressing could influence GC progression by different mechanisms.

## 4. GC-MSCs and Tumor Immunity

Accumulating evidence has demonstrated that TA-MSCs could affect tumor progression through the bidirectional interactions with innate and adaptive immune systems [34, 47, 65, 66]. Herein, we highlight the interactions between GC-MSCs and immune cells or molecules, and how they impact tumor progression, which are very interesting and meaningful for GC immunotherapy.

### 4.1. GC-MSCs and Innate Immunity

TA-MSCs have been reported to recruit innate immune cells, macrophages, and neutrophils in particular and interact with them to enhance the anti-inflammatory condition, thereby promoting tumor progression [23, 67–70]. Based on studies to date, there are few to no studies about the interaction between GC-MSCs and macrophages or myeloid-derived suppressor cells (MDSCs). Here, we mainly elucidate the communication between GC-MSCs and neutrophils. Zhu et al. first proved that bidirectional interactions between GC-MSCs and neutrophils exist in the GC environment, which could influence the GC progression [71]. They concluded that GC-MSC-CM remarkably prompted the chemotaxis of neutrophils and skewed them towards the activated state. Moreover, GC-MSC-CM could also inhibit the spontaneous apoptosis of neutrophils. Although GC-MSC-primed neutrophils have no function on GC cell proliferation, they promote the migration and angiogenesis of GC cells and could induce the transition of GCN-MSCs into CAFs. TA-MSCs were reported to have the ability to differentiate into *α*-SMA^+^ CAFs [72, 73]. And whether GC-MSC-primed neutrophils are able to induce GC-MSCs into CAFs, which also have a tumor-promoting role, need to be further investigated. Mechanistically, GC-MSC-CM-derived IL-6 mediated the activation of STAT3-ERK1/2 signaling cascade in neutrophils, regulating the above communication between GC-MSCs and neutrophils, and their impacts on the promigratory and proangiogenicity of GC cells. These results show the importance of bidirectional interaction between GC-MSCs and innate immunity, which could obviously influence tumor progression.

### 4.2. GC-MSCs and Adaptive Immunity

Emerging evidence has demonstrated that MSCs have immunosuppressive effects on the adaptive immune system, thereby enhancing tumor growth [74–76]. TA-MSCs are the crucial component of the TME and are more direct than BM-MSCs in the study of interactions among MSCs, immune cells, tumor cells, and other environmental cells. Wang et al. found that GC-MSC-CM could obviously reverse the inhibitory effects of peripheral blood mononuclear cells (PBMCs) on the GC growth *in vivo*, but not *in vitro* [77]. And GC-MSC-CM-educated PBMCs could significantly enhance the migration and EMT of GC cells *in vitro* and liver metastases *in vivo*. Moreover, GC-MSC-CM dampened Treg/Th17 balance in PBMCs through inhibiting Th17 cell proliferation and inducing Treg differentiation. Mechanistically, Sun et al. further proved that GC-MSC-derived IL-15 increased Tregs by activating STAT5 in CD4^+^ T cells, which enhanced GC cell migration [55]. Unexpectedly, they found that IL-15 also promoted PD-1 expression in Tregs, which was worth further investigating [55]. Interestingly, GC-MSC-derived IL-8 could regulate immune checkpoints, PD-L1 in GC cells through STAT3/mTOR-c-Myc signal axis [78]. And it also protects GC cells from the cytotoxic effect of CD8^+^ T cells by upregulating PD-L1 in GC cells [78]. As shown above, GC-MSCs can regulate the functionality of adaptive immune cells, thereby influencing tumor progression. Xu et al. proved that GC-MSCs are plastic, whose phenotype and immunomodulatory ability could be modified by adaptive immune cells [79]. CD4^+^ T cells could educate GC-MSCs with PD-L1 upregulation, which promoted GC cell migration and GC growth *in vivo* via PD-1/mammalian target of rapamycin (mTOR) signaling axis while did not impact apoptosis and proliferation of GC cells *in vitro* [79]. To sum up, bidirectional interactions between GC-MSCs and adaptive system cells greatly affect the GC progression, which may provide novel approaches for immunotherapy.

## 5. Biomarker and Therapy

Emerging studies have shed light on the diagnostic and prognostic potential of tumoral miRNAs in GC [80, 81]. Deregulated miRNA levels have been investigated clinically acting as diagnostic biomarkers in several biopsy specimens and body fluids [82, 83]. But few studies demonstrated the diagnostic and prognostic value of miRNAs in TA-MSCs. Wang et al. found that miR-214 in circulating levels and miR-221 and miR-222 in GC-MSC levels were significantly higher in the GC-MSC group than in the relevant GCN-MSC group, which was consistent with the differential expression levels between GC tissues and noncancerous gastric tissues [62]. They also proved the correlations, respectively, between miR-221 and miR-222 high levels and extensive lymph node metastasis, miR-214 and miR-222 high levels and serosal invasion, miR-221 and miR-222 high levels and the TNM stage, and miR-214 high levels and venous invasion [62]. In addition to miRNA, other molecules from GC-MSCs have the potential to act as a biomarker. The elevated serum IL-15, which was also mainly derived from GC-MSCs, was proved to be significantly correlated with lymph node metastasis in GC, but not with other clinicopathological parameters [55]. To summarize, GC-MSCs also have the diagnostic and prognostic value, which is crucial for clinical therapy.

Studies of the GC-MSC-associated therapy are rare, which means more efforts need to be made in this field. Huang et al. found a compound, named curcumin, suppressing GC-MSC-mediated angiogenesis through inhibiting NF-*κ*B/VEGF signaling axis, which means a novel therapeutic approach for GC progression via targeting GC-MSC-driven angiogenesis [84]. Interestingly, we also discerned that low level 3,3′-diindolylmethane (DIM) evidently enhanced GC cell proliferation and migration through activating wnt4 signaling while high level obviously inhibited GC growth, which provide a new strategy for the optimum concentration of DIM in its clinical application. Also, the effects of DIM on the functions of GC-MSCs need to be further investigated [85].

In summary, GC-MSCs could play the important biomarker and therapeutic role in GC progression, providing potentially novel therapeutic targets for tumor therapy.

## 6. Discussion and Perspectives

**Figure 1:**
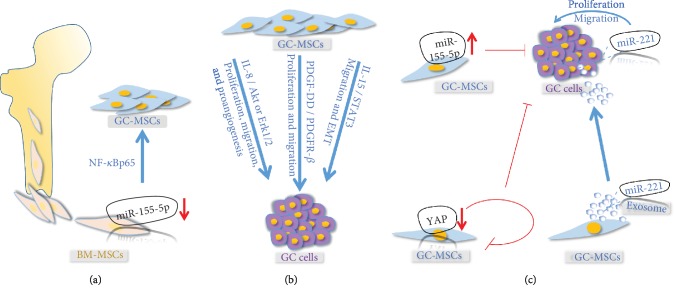
The important role of GC-MSCs in GC progression. (a) GC-MSC origin from BM-MSCs, which could be mediated by miR-155-5p inhibition to transfer into GC-MSCs. (b) GC-MSC-derived CM, containing molecules including IL-8, IL-15, and PDGF-DD, promote GC progression through AKT/ERK, PDGF-DD/PDGFR-*β*, IL-15/STAT3 signaling pathways, respectively. (c) Changes of molecule expression in GC-MSC influence GC progression. miR-155-5p overexpression in GC-MSCs could reverse the tumor-promoting phenotype and function; YAP knockdown in GC-MSCs inhibits GC-MSC functionality and suppresses the GC growth; miR-221 in GC-MSCs could be delivered to GC cells through exosomes, which increase miR-21 expression in GC cells and promote GC progression.

**Figure 2:**
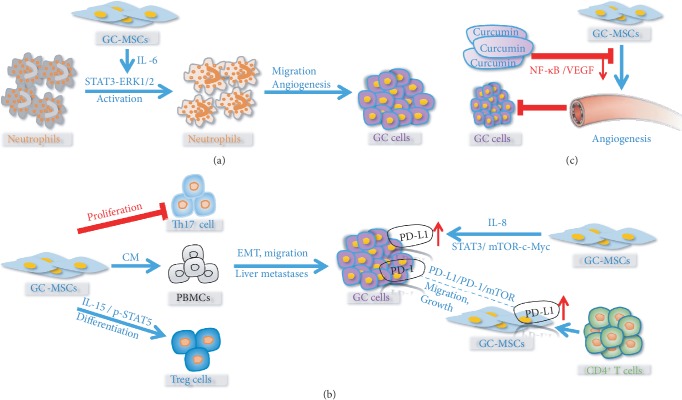
The interaction between GC-MSCs and immune cells and therapy. (a) GC-MSC-derived IL-6 induces the activation of neutrophils through STAT3-ERK1/2 signaling axis, promoting migration and angiogenesis of GC cells. (b) GC-MSC-CM inhibits the proliferation of antitumor immune cells, Th17 cells. GC-MSC-derived IL-15 can promote the differentiation of protumor immune cells, Tregs through activating STAT5. GC-MSC-educated PBMCs could also induce migration and the EMT of GC cells. GC-MSC-derived IL-8 could promote PD-L1 expression in GC cells through STAT3/mTOR-c-Myc signaling axis. CD4^+^ T cells educate GC-MSCs with PD-L1 upregulation, promoting GC cell migration and GC growth via activating PD-1/mTOR signaling. (c) Curcumin could inhibit GC-MSC-mediated angiogenesis and suppress GC growth.

Diverse potential abilities of MSCs are constantly being excavated, from its crucial role in protumor and antitumor progression to recently several novel MSC-mediated therapies [73, 86–90]. TA-MSCs, as the critical component of TME, are concerned by more and more studies. They interact with microenvironmental cells more directly than other MSC type, so it is meaningful to deeply investigate the further function of TA-MSCs. Tumor immunity could obviously influence tumor fate. It is easier to combine with the clinic and has a better clinical application value, which is a well-being of tumor treatment [91–95]. So, correlations between TA-MSCs and tumor immunity need to be given more attention and investigated, which is promising and could offer a blueprint for tumor immunotherapy. In this review, we mainly elucidate the essential and indispensable role of GC-MSCs in the GC progression from tumor to tumor immunity, which has the potential therapeutic value (Figures 1 and 2, Table 1). And thought-provoking in the above GC-associated studies, we would discuss here. PD-1 and PD-L1 pair, the leading immune checkpoint pathway in the TME, plays a immunosuppressive role by suppressing the function of T cells and tumor-infiltrating lymphocytes, resultingly promoting tumor progression [96, 97]. Owing to the role of GC-MSC-derived IL-8 and the function of CD4^+^ T cell-primed GC-MSCs with PD-L1 upregulation we have described [54, 78, 79], we speculate that PD-L1 expression in GC-MSCs could affect IL-8 secretion and mediate the GC progression, which we are investigating recently. GC-MSC-CM, CD4^+^ T cell-primed GC-MSC-CM and GC-MSC-primed neutrophils, had no impact on the proliferation of GC cells in vitro, but all of them promote GC growth in vivo [71, 79], which remind that the underlying microenvironmental cells or mechanisms work. Whether the proangiogenic role of GC-MSCs only works *in vivo* need to be further investigated [98]. Moreover, the interactions between GC-MSCs and other immune cells including B cells, macrophages, NK cells, and MDSCs need to be further investigated, which are more valuable for deeply exploring the correlation between GC and immune systems and the effectively clinical immunotherapy.

## Figures and Tables

**Table 1:** The emerging role of GC-MSCs in the gastric cancer microenvironment.

Critical molecules/cells	Effect	Reference
PDGF-DD	GC-MSC-derived PDGF-DD promoted GC cell proliferation and migration by PDGF-DD/PDGFR-*β* signaling pathway.	[53]
IL-8	GC-MSC-derived IL-8 enhanced the proliferation, migration, and proangiogenesis ability of GC cells partly by regulating the activation of Akt or Erk1/2 pathway.	[54]
IL-15	GC-MSC-derived IL-15 could promote GC cell migration and epithelial-mesenchymal transition (EMT) by regulating STAT3 in GC cells.	[55]
miR-221	GC-MSC-CM-derived exosome carrying miR-221 regulated the proliferative and migratory ability of GC cells.	[62]
miR-374	miR-374 participated in regulating gastric carcinogenesis.	[63]
miR-155-5p	miR-155-5p inhibition could regulate the transition of BM-MSC into GC-MSC-like cells.	[49]
YAP	YAP expression in GC-MSCs can affect GC growth and progression in vitro and in vivo.	[64]
Neutrophils	GC-MSC-CM remarkably prompted the chemotaxis of neutrophils and skewed them towards the activated state through GC-MSC-CM-derived IL-6 that mediated the activation of STAT3-ERK1/2 signaling in neutrophils, which could promote migration and angiogenesis of GC.	[71]
PBMCs	GC-MSC-CM could obviously reverse the inhibitory effects of peripheral blood mononuclear cells (PBMCs) on the GC growth. And GC-MSC-CM dampened Treg/Th17 balance in PBMCs.	[77]
